# Assessment of management styles among top nursing leaders in Slovenian primary health centers: a cross-sectional analysis

**DOI:** 10.1108/LHS-10-2023-0083

**Published:** 2024-02-27

**Authors:** Melita Peršolja, Boštjan Žvanut, Špela Rot, Mirko Markič

**Affiliations:** Faculty of Health Sciences, University of Primorska, Izola, Slovenia; Primary Health Center Logatec, Logatec, Slovenia; Faculty of Management, University of Primorska, Koper, Slovenia

**Keywords:** Management, Nursing leadership, Nurse managers, Primary nursing

## Abstract

**Purpose:**

This study aims to endeavor to discern the predominant leadership styles used by nursing managers within the framework of Slovenian primary health centers. Using a quantitative research approach, the study was conducted through the administration of a structured questionnaire.

**Design/methodology/approach:**

The investigation encompassed 67 nursing managers, representing the entire spectrum of primary health centers in Slovenia. A stratified representative subset comprising 53 top nursing managers actively participated in this study.

**Findings:**

The prevailing leadership style among nursing managers predominantly manifests as the “integrated” style, characterized by a balanced emphasis on both interpersonal relationships and task-oriented elements. These nursing leaders exhibited a proclivity for fostering collaborative teamwork, with their leadership approach notably shaped by traits such as positive thinking, self-assuredness, comprehensive leadership knowledge and an intrinsic motivation to guide and inspire individuals. Notably, leadership knowledge emerged as the most influential factor in determining the selected leadership style. The study’s findings recognize specific areas in which leadership competencies among nurse managers may require further enhancement and development.

**Originality/value:**

The study’s findings are based on a specific subset of nursing leaders in a particular region, which can add to the originality, especially as there is limited prior research in this specific context. The study’s exploration of leadership styles is original in the sense that it provides insights into the leadership behaviors and traits of nursing managers in the given context. The emphasis on factors such as positive thinking and leadership knowledge as influential elements adds originality to the study.

## Introduction

Leadership style encompasses a multifaceted array of behaviors that exert an impact on interactions between leaders and colleagues. The aptness of a leadership style is intricately tied to the context, wherein the specific situation operates as the principal determinant guiding the choice of style (Blanchard *et al.*, 2013). Numerous studies (Anselmann and Mulder, 2020; Warri, 2021; Wong *et al.*, 2013; Wong and Giallonardo, 2013; Wong, 2015) have highlighted the significant impact of managers and their leadership styles on the quality of nursing services.

Effective leadership contributes to the enhancement of professional autonomy and clinical management. It also eases adaptations in management practices, orienting them more toward patient needs and aligning them with the requirements of hierarchical administrative systems (Jodar *et al.*, 2016). The leadership style adopted by nurse managers holds substantial influence over nurse satisfaction, turnover rates and the overall quality of patient care they deliver (Saleh *et al.*, 2018). For instance, in a study by Adams *et al.* (2018), the relationship between patient falls resulting in injury and a leader’s character traits, strategies, solutions and expectations of their staff was found to be notably strong.

The relationship between relationship-based management and the reduction of adverse events, particularly medication administration errors, patient mortality, nosocomial infections and pressure ulcers, has also been shown to be critical (Akbiyik *et al.*, 2020; Wong *et al.*, 2013). According to Akbiyik *et al.* (2020), relationship-oriented leadership behaviors improve patient outcomes and improve the quality of nursing care compared to task-oriented leadership behaviors.

## Background

The four leadership styles proposed by Reddin (1967) represent distinct approaches to leadership within an organizational context. These styles have been the subject of extensive research and analysis in the field of leadership studies, providing valuable insights into how leaders can adapt their behaviors to effectively lead teams in various situations.

In the related leadership style, leaders place a high value on developing and maintaining strong interpersonal relationships within their teams. This approach is rooted in social exchange theory, which suggests that individuals are more likely to cooperate and work effectively when they perceive positive and supportive relationships with their leaders. Effective implementation of this style may result in higher levels of team cohesion, trust and collaboration, which in turn can lead to improved performance and job satisfaction.

Integrated leadership style emphasizes teamwork, open communication and a forward-looking orientation, which encourages employees to actively contribute to decision-making processes. Leaders who foster open communication and collaboration create an environment conducive to idea exchange and problem-solving, ultimately promoting organizational learning and growth. This leadership style is often associated with high levels of employee motivation, as team members feel empowered and engaged in shaping the future of the organization.

Dedicated leadership style involves leaders who assertively take charge, providing clear direction and making explicit demands on team members to accomplish tasks and goals. This style draws from the concept of task-oriented leadership, where leaders provide structure and closely monitor performance. Dedicated leadership can be particularly effective in situations requiring swift decision-making and crisis management. When leaders are decisive and directive, it can instill a sense of discipline and accountability within the team. However, it is important for leaders using this style to strike a balance, as overemphasis on control may lead to employee dissatisfaction and resistance.

Separated leadership involves leaders who adhere to established policies and rules, enforcing them rigorously to ensure strict compliance within the organization. This style reflects an autocratic or bureaucratic leadership approach, where leaders place a premium on conformity and adherence to established norms. Separated leadership can be effective in highly regulated and compliance-driven industries, such as health care, where strict adherence to standards is essential for minimizing risks. However, this style may foster employee creativity, innovation and adaptability, as it tends to emphasize conformity over flexibility and agility.

Reddin's four leadership styles offer valuable frameworks for understanding how leaders can adapt their behaviors to suit different organizational contexts and objectives. Each style has its own unique strengths and limitations, and their effectiveness depends on the specific demands of the situation and the needs of the team and organization. As Reddin (1970) explains, any leadership style can be effective or ineffective. Effective leadership is contingent upon the harmonization of the leadership style with specific situational requirements. When leadership style aligns with these demands, it is considered effective; conversely, a mismatch is indicative of ineffectiveness. The degree of effectiveness or ineffectiveness is contingent upon the unique attributes of the situation, encompassing task complexity, employee characteristics, interactions with colleagues, relations with superiors and the prevailing organizational structure. Top nursing managers are expected to possess the proficiency to transition seamlessly between diverse leadership styles. This adaptability is particularly crucial in the realm of nursing, given its multifaceted nature, spanning both creative and routine tasks.

The idea is that effective leaders can adapt their style based on the task and the readiness of their team members to take on responsibilities. The model suggests leaders should adjust their style based on the situation, the capabilities of their team and the nature of the task at hand.

While nursing's origins were rooted in the care of individuals, contemporary nursing practice has shifted toward an increasing focus on tasks involving data rather than direct patient care (Peršolja, 2021). Historically, nurses preferred leaders who embodied qualities such as articulateness, proactivity and independence in nursing leadership (Azaare and Gross, 2011). However, the evolving landscape of health care, marked by rapid organizational change, needs a fresh style of leadership (Jouany and Martic, 2020). First-line nurse managers hold indispensable roles within health-care organizations. Their responsibilities are multifaceted, essential and often intricate (Paarima *et al.*, 2022). The primary tasks of these managers involve ensuring the provision of high-quality and safe care (Ofei *et al.*, 2018) while acting as intermediaries between top-level management and nursing staff. They are responsible for fostering and sustaining healthy and secure work environments, which positively impact both staff and patient outcomes, thereby reducing mortality rates across all health-care systems (Alomairi *et al.*, 2018). This pivotal role encompasses varying degrees of rigor and breadth (Erjavec and Starc, 2017), necessitating frontline nurse managers to possess the requisite leadership skills to ensure efficiency and effectiveness within their units. The enhancement of nursing leadership skills calls for a shift toward behaviors underpinned by greater respect, a commitment to the well-being of others, ongoing professional development and the expression of appreciation (Morsiani *et al.*, 2017). To enhance leadership in health care, we require solid support from health-care organizations and a well-organized educational framework (Kiwanuka *et al.*, 2021), especially because of the increasing demand for leaders with multidimensional competencies within health-care systems (Ocho *et al.*, 2021).

## Aims

Considering the substantial progress achieved in research related to managerial styles within the domain of hospital nursing, a conspicuous gap becomes apparent in the existing body of literature when considering the management practices within primary health centers. Also, though Reddin's model has been widely used, it has not been well-tested by primary health center nursing managers. This study’s main aim was therefore to give a full description of the self-perceived leadership styles among nursing managers within primary health centers.

We have formulated the following research inquiries to address this void: What is the predominant leadership style shown by nursing managers in primary health centers? Which personal attributes and characteristics wield a pivotal influence in shaping the leadership style of these managers?

## Methods

### Design

A non-experimental, descriptive research method was used. A cross-sectional survey was conducted using a closed-ended questionnaire.

### Instrument

We used the Reddin’s management styles questionnaire (Reddin, 1970), which is typically used in organizational and leadership development contexts, to assess and understand a leader’s preferred management style. It can be applied in several situations, including: leadership development programs, management training, team building, conflict resolution, succession planning and change management. It is a tool for self-awareness and enhancing leadership effectiveness. The tool was translated into Slovenian by Kaučič (2009).

The instrument held three sections. The first pertained to characteristics of the respondents, such as gender, education, age, years of work experience, years employed in the current organization, job title, the number of employees they supervise, whether the position of Head Nurse is tied to a mandate, whether they are involved in direct patient care and whether they received functional training for management and leadership before taking up the position.

The second section consisted of 17 statements that focused on leadership styles. The first seven statements relate to the integrative leadership style, and the following five relate to the reserved style. The assertive leadership style was defined by statements from 13 to 18, and the proactive leader was characterized by statements from 19 to 23. The third section comprised 16 statements that were designed to assess the effectiveness of leadership and gauge how leaders evaluate their personality traits (self-perception aspect). The respondent evaluates the statements using the following values: 1, if the statement is not true; 2, if the statement is somewhat true; 3, if the statement is mostly true; and 4, if the statement is entirely true.

The Cronbach’s coefficient for the questionnaire used in the leadership models section varies between 0.609 and 0.969. For the leadership styles section, it is 0.636, and for leadership effectiveness, it is 0.866. These numbers reflect that the measurement tool we used is reliable.

### Population and sample

The Slovenian health-care system is organized as a universal, publicly funded system that provides comprehensive health-care coverage to all its residents. It follows a model of compulsory health insurance, where residents are required to have health insurance coverage. The system consists of primary, secondary and tertiary levels of health care. Primary care serves as the initial point of contact for patients and is usually provided by general practitioners or family doctors. Secondary health care involves specialized care and is provided in hospitals and specialized clinics. Patients are referred to secondary care by their primary care physicians when more specialized treatment or consultations with specialists are required. Tertiary care encompasses highly specialized and complex medical services, often provided in specialized hospitals or university medical centers. These facilities handle severe or rare conditions and offer specialized treatments not available at lower levels of care. Overall, the Slovenian health-care system aims to provide accessible and high-quality health-care services to all citizens.

The data were collected between June and November 2020. The nursing managers of all 67 Slovenian public health centers at the primary health care level were invited to participate in this study. The questionnaire with the corresponding invitation letter was sent to the participants by email or post. Because of the low initial response rate, we had to re-invite the nursing managers to participate in the study and/or contact them personally. The final sample consisted of 53 participants (response rate 79.1%).

Most participants were female (81.1%) and had a graduate degree, but no training for a management position (Table 1). The average age of the participants was 46.4 years (SD *= 8.2*). On average, they had 25.0 years of experience (SD = 8.1), 18.6 years in their current facility (SD = 8.8) and 8.9 years in their current position as a nursing manager (SD = 6.3). They were responsible for 20–800 employees (*M* = 87.4, SD = 107.7) and, on average, worked 12.6 (SD = 7.0) hours per week with patients.

### Data analysis

First, univariate data analysis was performed to calculate the mean (*M*), standard deviation (SD), median (Me) and Quartiles 1 and 3 (Q1, Q3). In addition, the normality of the distribution of the numerical variables was assessed by analysing the corresponding histograms and calculating the Shapiro–Wilk test. Cronbach’s alpha coefficients were calculated to assess the internal consistency of the participants’ responses.

The leadership style indexes were calculated as the mean values of the corresponding items (integrated leadership style: items 1–8; separated: items 9–14; related: items 15–21; dedicated leadership style: items 22–27).

In addition, a Wilcoxon signed-rank test was used in post hoc tests. Furthermore, multiple linear regressions (forward) were performed to name the predictors of each leadership style. All assumptions of multiple linear regressions were carefully considered (e.g. homoscedasticity, absence of multicollinearity, constant variance and normal distribution of residuals). The statistical significance level was set at 0.05. Bonferroni correction was applied when evaluating the results of post hoc tests.

### Ethical considerations

Institutional review board approval was obtained prior to the start of the study from all primary health centers in Slovenia. The study was conducted following the Code of Ethics for Nurses and Nurse Assistants (Kodeks etike v zdravstveni negi in oskrbi, 2014), as well as the Helsinki Declaration (World Medical Association, 2013). The nurse’s managers were informed that “research on management” was being conducted, provided with information about what would happen to the data collected, and offered the name and details of the responsible contact when necessary. Consent from nursing managers for their participation in the study was procured in-person at the study sites. Furthermore, respondents who engaged with the online questionnaire were required to provide their explicit consent for participation prior to completing the questionnaire.

## Results

### Leadership styles

Table 2 shows the results of descriptive statistics for Reddins' leadership style indexes, where the integrated leadership style had the highest average value, while the lowest was for the separated style. The result of Friedman’s test shows statistically significant differences in the average values of ranks of each leadership style index (*χ*^2^(3, *N* = 53) = 120.417, *p* < 0.001). The self-assessed leadership style was not significantly correlated to any demographic characteristics of nursing managers.

### Personal characteristics

A descriptive analysis of self-assessed leadership attributes (as presented in Table 3) reveals that several attributes are of particular significance. These include the capacity to foster open and constructive relationships, the willingness to confront challenges while seeking optimal pathways toward objectives, a dedicated pursuit of excellence in the nursing domain and an active pursuit of shared goals with colleagues.

Conversely, managers assigned the lowest ratings to statements suggesting that leadership roles are integral to their personal lives, that they possess substantial expertise in leadership and motivation, that they possess key competencies associated with effective leadership, and that they perceive themselves as highly successful leaders.

### Predictors of leadership style

Multiple regressions (forward) were run to determine if the leadership characteristics were predictors of different leadership style indexes. The results in Table 4 show the models of the last iteration (adjusted *R*^2^ and statistical significance of the model – *p*) and the identified predictors with the standardized coefficients β and *p* values.

In the first model, three leadership characteristics, i.e. knowledge; trust and positivity; and motivation, appeared as positive and significant predictors of the 3D leadership style index and explained 35.1% of its variance. In the second model, interpersonal relationships, trust and positivity were found to be positive and significant predictors of the related leadership style index, explaining 45.6% of its variance (large effect). In the third model, knowledge and working at home proved to be positive and significant predictors of the separated leadership style index, explaining 25.7% of its variance (medium effect). In the fourth model, only the variable interpersonal relationships proved to be a positive and significant predictor of the integrated leadership style index, explaining 20.3% of its variance (medium effect). No leadership characteristics were identified as significant predictors of the dedicated leadership style index, and therefore it is not presented in Table 4.

Our aim was to investigate the presence of a statistical correlation between one's ability in the realm of personnel management and their intrinsic motivation for assuming leadership responsibilities. To this end, we conducted a correlation analysis, which showed that variables are significantly associated (*r* = 0.444, *p* = 0.001, *n* = 53). Positive thinking, high self-confidence, profound expertise in leadership and motivation, problem-solving capabilities and a commitment to achieving excellence in health care are quantifiable factors that demonstrate an association with successful leadership capacity (Table 5).

## Discussion

The survey encompassed a representative sample of nursing managers in primary health centers across Slovenia, and the predominant leadership style observed was the integrated style. We can assume that our sample reflects the population under consideration.

It is a matter of concern that the majority of top nursing managers in our study appear to rely on their instincts for leadership, as only seven of them received formal training in management and leadership. The ideal top nursing managers are expected to possess a well-rounded knowledge of various management styles, enabling them to select the most suitable style based on the unique characteristics of the organization and external circumstances. Effective nursing top managers are characterized by their proficiency in people-oriented, task-oriented and efficiency-driven leadership. They set ambitious objectives, demand high-performance standards, nurture positive relationships and remain receptive to innovative concepts. Modern leadership paradigms necessitate organizational support and training, as outlined by Kiwanuka *et al.* (2021). The education and cultivation of successful and effective leaders stand as both a professional and moral responsibility of top nursing managers, as emphasized by Specchia *et al.* (2021).

The style chosen shows that nursing managers focus on relationships as well as tasks. They are advocates of teamwork and identify with their subordinates, and thus desire partnership rather than a strict hierarchy. They do not use managerial hierarchy in leadership, as this would slow down the introduction of change and innovative ideas. Nursing managers also strive to have open interpersonal relationships, accept challenges and look for the best ways to reach their goals. This relational leadership creates a trusting environment that contributes to a better quality of care (Akbiyik *et al.*, 2020; Kiwanuka *et al.*, 2021). Nursing is people-oriented, and thus it is important to encourage top nursing managers to engage in collaborative leadership behaviors (O'Donovan *et al.*, 2021). Effective communication is consistently highlighted in research as an essential skill for a leader (Zor Šabič, 2016). Better outcomes in nursing have also been proven when employees perceive the leader as professional, honest and transparent, with strong ethics, integrity, support, understanding and problem-solving skills (Saleh *et al.*, 2018). Empowering and relational leadership styles are associated with positive outcomes for nursing team performance (O'Donovan *et al.*, 2021).

The leader should be able to choose the right leadership style to achieve the organization's goals in each situation. All four leadership styles mentioned by Reddin (1967) were found in our research population, but the most common was the integrated style. Leadership style was predicted by some personal characteristics but not by any demographic ones. The results are consistent with those of other researchers such as Kramar (2016) and Mezek (2011) and could be explained by the fact that individual behavior always transcends gender (and other demographic characteristics) and considers other factors such as a person’s physical environment, heredity, culture and experiences (Lega, Prenestini and Rosso, 2017; Apore and Asamoah, 2019).

Our investigation unveiled that the leadership style adopted by top nursing managers is significantly associated with specific factors, notably positive thinking, self-confidence, leadership knowledge and the motivation to guide and inspire individuals. Our findings align with previous research by Mohd-Shamsudin and Chuttipattana (2012), who demonstrated that leadership competency can be anticipated through an interplay of personality traits and motivation, with motivation often exerting a more prominent influence than personality. However, it is worth noting a contrasting outcome in our study, where leadership knowledge emerged as the predominant predictor of the chosen leadership style. This underscores the pivotal role of enhancing and nurturing leadership competencies.

### Limitations

There are many limitations that should be considered before generalizing the results of our study. First, the results of this study are based on participants’ self-assessment, which may lead to socially biased responses. Additionally, to avoid this bias, the participants were asked to supply honest responses to provide a realistic picture in this field. Second, this study is limited to one country; however, our findings can be generalized to other countries in the region (i.e. Croatia, Serbia, Bosnia and Herzegovina) that share a common history when health centers were developed and have a similar organization of nursing in health centers. The third limitation is that in this study, leadership styles were not associated with measurable (frequency of pressure ulcers, number of falls, MRSA infections, etc.) and non-objectively measurable (e.g. patient satisfaction, employee satisfaction, founders, clinical pathways, etc.) management effectiveness indicators.

### Recommendations for further research

Hence, it is evident that additional research is warranted to delve deeper into the realm of leadership styles among top nursing managers in primary health centers, with a specific focus on their implications for employee and patient satisfaction, as well as the attainment of quality standards. Of particular interest is the exploration of the intricate relationship between leadership styles and variables such as commitment and dedication, given that leadership styles alone may not exert a direct impact on organizational performance. In addition, the areas of absenteeism, presentism and workforce turnover represent crucial facets deserving of extended investigation in relation to leadership styles.

Moreover, it would be of significant value to extend such inquiries to tertiary health-care settings, facilitating a comparative analysis across distinct management levels within nursing care organizations. To effectively track and comprehend the evolving landscape of nursing leadership styles, we recommend the periodic repetition of surveys at intervals spanning three to five years. This longitudinal approach will enable us to monitor and decipher trends in leadership styles within the nursing management domain.

## Conclusion

The existing body of literature has been notably deficient in shedding light on the leadership styles adopted by top nursing managers within the primary health-care domain. Our study effectively addressed this gap by revealing that, within Slovenian primary health centers, top nursing managers predominantly use an integrated leadership management approach, characterized by their balanced emphasis on both interpersonal relationships and task-oriented aspects. Notably, our analyses established that personal attributes, rather than demographic variables, significantly influence the selected management style. Among these attributes, positive thinking and self-confidence emerged as the most influential predictors.

These findings hold substantial implications for decision-makers, emphasizing the pressing need to bolster specific leadership competencies among nurse managers. Effective leadership necessitates an astute understanding of situational variables, which enable leaders to select the most pertinent style for accomplishing organizational objectives within distinct contexts.

With shifting demographics and evolving patient demands, health-care systems both domestically and internationally could benefit significantly from this research. This paper emphasizes the crucial necessity for nursing managers to undergo comprehensive preparation and specialized education tailored specifically to their role. The insights gleaned from this study are poised to be instrumental in driving personnel development initiatives, particularly in the formulation of robust management training and development programs. These programs, informed by our findings, can play an instrumental role in honing the leadership skills of nursing managers and enhancing their efficacy in health-care management roles.

## Figures and Tables

**Table 1.** Participants’ demographic data – descriptive statistics (*n* = 52)

Variable	*n*	%
*Gender*		
Female	43	81.1
Male	10	18.9
*Level of education*		
Vocational college*	4	7.5
BSc	23	43.4
University study program	25	47.2
Specialization, MSc	1	1.9
*Mandate*		
Not tied to a mandate	24	45.3
Two years	1	1.9
Four years	27	50.9
Five year	1	1.9
*Direct contact with patients*		
Yes	31	58.5
No	22	41.5
*Training in leadership/management before occupying the manager position*		
Yes	7	13.2
No	46	86.8

Notes:

*Particularity of Slovenia – a two-year study program in the period 1954–1996; *n* = number of samples; *M* = mean value; SD = statistical deviation

**Table 2.** Reddins’ leadership style indexes – descriptive statistics (*n* = 53)

Index of leadership styles	*M*	SD	Me	(Q_1_, Q_3_)
Integrated	4.34	0.36	4.38	(4.1, 4.5)
Related	4.15	0.32	4.14	(4.0, 4.3)
Dedicated	3.15	0.57	3.00	(2.8, 3.5)
Separated	2.65	0.54	2.67	(2.3, 3.0)

Notes:

*M* = mean value; SD = standard deviation; Me = median; Q_1_ = Quartile 1, Q_3_ = Quartile 3

**Table 3.** Nursing managers’ personal characteristics – descriptive statistics (*n* = 53)

Item	*M*	SD	Me	(Q_1_, Q_3_)
I accept challenges and look for the best paths to the goal	4.7	0.5	5	(4, 5)
I maintain open interpersonal relationships	4.7	0.4	5	(4, 5)
As a leader, I strive for advances in nursing	4.6	0.7	5	(4, 5)
I’m very motivated to lead people	4.5	0.6	5	(4, 5)
I achieve my goals together with my colleagues	4.5	0.6	5	(4, 5)
I can analyze and solve the problems	4.4	0.6	4	(4, 5)
I clearly define goals and accept the risk	4.4	0.6	4	(4, 5)
I think positively and have considerable confidence in myself	4.4	0.6	4	(4, 5)
I have the ability to learn from problems at work	4.4	0.6	4	(4, 5)
I take career problems as an opportunity and as a source from which I can learn	4.3	0.6	4	(4, 5)
I’m ambitious	4.2	0.7	4	(4, 5)
I’m ready to work at home after work	4.1	1.0	4	(4, 5)
I have the key skills to be a successful leader, and I’m a successful leader	4.0	0.8	4	(4, 5)
I have a lot of knowledge about leading and motivating people	3.9	0.8	4	(3, 4)
I have a desire for great achievements	3.9	0.7	4	(3, 4)
The work of a leader is an important element of my life	3.7	0.8	4	(3, 4)

Notes:

*M* = mean value; SD = standard deviation; Me = median; Q_1_ = Quartile 1, Q_3_ = Quartile 3

**Table 4.** Multiple regression analysis results (forward)

Model no.	Dependent variable	Model summary		Predictors		
		Adjusted *R*^2^ (%)	*p*	Variable	*β*	*p*
1	INDEX_4D	35.1	<0.001			
				Knowledge about leading and motivating	0.287	0.035
				Positivism, confidence	0.264	0.038
				Motivation to lead	0.260	0.044
2	Related leadership style index	45.6	<0.001			
				Open interpersonal relationship	0.534	<0.001
				Positivism, confidence	0.323	0.004
3	Separated leadership style index	25.7	<0.001			
				Knowledge about leading and motivating	0.480	<0.001
				Working home after work	0.266	0.031
4	Integrated leadership style index	20.3	<0.001			
				Open interpersonal relationship	0.467	<0.001

Notes:

% = percent; *p* = statistical significance; *β* = beta coefficient; INDEX_4D = constructed variable, including all four leadership styles

**Table 5.** Factors predicting successful leadership

Factor	Value					
	*B*	SD	*β*	*t*	*p*	*R* ^2^
Positive thinking and high self-confidence	0.515	0.178	0.370	2.886	0.006	55.9
Commitment to achieving excellence in health care	0.344	0.146	0.292	2.351	0.023	
Profound expertise to lead and motivate	0.231	0.128	0.230	1.802	0.078	
Have problem-solving capabilities	0.207	0.168	0.150	1.235	0.223	

Notes:

B = B value; SD = standard deviation; β = beta; *t* = t-value; *p* = statistical significance; R^2^ = R-squared
